# The Attitude Toward Seasonal Influenza Vaccine Among Workers of Community Healthcare Centers in Zhejiang Province, China: Barriers and Facilitators

**DOI:** 10.3390/vaccines13050507

**Published:** 2025-05-12

**Authors:** Jianyong Shen, Shangyan Han, Chao Zhang, Huakun Lv, Yu Hu

**Affiliations:** 1 Institute of Immunization and Prevention, Huzhou Municipal Center for Disease Control and Prevention, Huzhou 313000, China; hzcdcssjy@163.com (J.S.); zc5114348005@126.com (C.Z.); 2 Institute of Immunization and Prevention, Zhejiang Provincial Center for Disease Control and Prevention, Hangzhou 310051, China; yyybcxc@163.com (S.H.); hklv@cdc.zj.cn (H.L.); 3 School of Public Health, Hangzhou Medical College, Hangzhou 310053, China

**Keywords:** influenza vaccination, community healthcare center, vaccination compliance, vaccination coverage, attitude

## Abstract

**Background:** This study was aimed at understanding the attitude on influenza and influenza vaccination among workers of community healthcare centers (CHCs) and investigating the barriers and facilitators for influenza vaccination. **Methods:** A cross-sectional study was conducted through an anonymous questionnaire to all workers of CHCs in 22 CHCs. Socio-demographic characteristics, reasons for acceptance or refusal of influenza vaccination, influenza vaccination status, and attitude toward influenza vaccination were collected. Suggested strategies for improving influenza vaccine uptake were also surveyed. Descriptive analyses were conducted depending on the distributions of variables. A logistic regression analysis was implemented to examine the association between influenza vaccination status in the 2022/2023 season and the potential predictors. The adjusted odds ratio (AOR) with 95% confidence interval (CI) was calculated. **Results:** In total, 2205 workers of CHCs participated in this study. Influenza vaccination coverage in the 2022/2023 season was 1.36%. The reason “To avoid influenza” met with the highest level of agreement for acceptance of influenza vaccination (median = 4.36 for 1–5-point Likert scale), while the reason “Not a high-risk group of influenza and possible complications” met with the highest level of agreement for refusal of influenza vaccination (median = 3.72 for 1–5-point Likert scale). The influenza vaccination status was significantly related to professional categories, regular exercise habits, sources of information on influenza vaccination, and attitude on recommending influenza vaccination to patients. The free influenza vaccination and mandatory vaccination policies were the most frequent suggestions for improving influenza vaccination coverage. **Conclusions:** A lower influenza vaccination coverage was observed in workers of CHCs, and it might be attributed to several risk factors. It was urgent to take actions on improving their understanding of, awareness of, and confidence in influenza vaccination. Free influenza vaccination and vaccination requirement policies might be helpful for enhancing vaccine uptake, especially for physicians and other healthcare workers.

## 1. Introduction

Seasonal influenza is a respiratory infection that occurs worldwide, with a significant impact on the public health. Although influenza is usually self-limiting at the population level, seasonal influenza causes considerable morbidity and mortality, having serious social and economic consequences. The World Health Organization (WHO) estimates that 10% of adults and 25% of children have influenza, of which 3–5 million develop a severe infection and 290,000–650,000 die of influenza annually [1]. Furthermore, large numbers of mild to moderate cases pose a significant burden to society, generating associated medical costs, causing social disruption, and reducing the workforce’s productivity. Molinari estimated that the total cost related to seasonal influenza was approximately USD 87 billion, of which USD 10 billion was the direct medical cost [2]. China has a significant burden of influenza-related diseases as we have a larger population of over 1.4 billion. Wang et al. estimated that the mean incidence of influenza was 6.48 per 1000 person per season between 2010 and 2020 in mainland China [3]. Li et al. estimated the influenza-associated all-cause mortality was 14.33 per 100,000 persons for the general Chinese population [4]. The total economic burden related to influenza in China was almost USD 3.8 billion in 2019, accounting for 0.27% of the GDP in that year [5].

Workers of community healthcare centers (CHCs) face a significantly higher risk of exposure to influenza in daily work, making them a priority group for influenza vaccination by the WHO and ministry of health in many countries [6]. There are two reasons for that recommendation. It is notable that the workers of CHCSs would be more likely to be infected from an infected patient due to their work environment, especially those in high-risk departments such as outpatient and emergency departments, leading to absenteeism and causing disruptions in medical services. A systematic review indicated that the incidence of lab-confirmed influenza in healthcare workers without vaccination was 3.4 times higher than that among the general unvaccinated adults [7]. Moreover, the possibility of transmission is bi-directional, and a “healthworkers to patient” transmission model is also possible, which would pose a greater risk of nosocomial influenza transmission to the other patients and spreading influenza to families or communities [8].

Influenza vaccination is considered as safe and effective for preventing influenza and its complications. A systematic review indicated that influenza vaccine could prevent 59% of laboratory-confirmed influenza and reduce 42% of influenza-like illness [9]. Various published studies did not provide enough evidence to warrant the introduction of mandatory influenza vaccination for healthcare workers, but scientific data support their voluntary vaccination [10,11]. For example, a randomized controlled trial conducted at hospitals demonstrated that vaccinated healthcare workers had a lower incidence of acute respiratory illnesses than unvaccinated healthcare workers (28.7% vs. 40.6%) and fewer days of sick leave (9.9% vs. 21.1%) [12].

In Zhejiang province, healthcare systems offer influenza vaccination as a self-paid vaccine for all workers of CHCs. The influenza vaccination was provided at vaccination clinics set in the CHCs, which are the most basic medical and health service institutions. The vaccination coverage of influenza among workers of CHCs had never been investigated in Zhejiang province, but we found it remained low in Europe [13]. The main reasons for low compliance had been investigated elsewhere to date, which included concerns of adverse reactions, vaccine effectiveness, and consideration of low risk of contracting influenza [14,15]. On the other hand, the positive reasons for motiving healthcare workers to accept influenza vaccination included protection for themselves and their patients, awareness of their role in the transmission of influenza, and convenient access to vaccination [16,17]. Other factors like profession category and education degree also impacted the vaccination coverage among healthcare workers [18]. Unfortunately, previous reported interventions to improve the influenza vaccination coverage mainly focused on leadership, the involvement of all parties concerned, reminders, peer pressure, social media, and a mandatory vaccination policy, which had generally been unsuccessful [19].

Influenza patients have a higher probability for seeking medical care firstly from primary care hospitals like CHCs in Zhejiang province, as the early symptoms of most cases are unspecified and mild. However, the prodromal stage of influenza is most contagious, sometimes at the peak of infectivity [20]. As such, improving the influenza vaccination coverage in CHCs would be one of the key control activities to protect healthcare workers and other non-medical workers of CHCs and decrease work absenteeism, as well as to prevent nosocomial infection of influenza and to reduce the morbidity and mortality at the community level.

This study was aimed at understanding the attitude on influenza and influenza vaccination among workers in community healthcare centers in Zhejiang province, as well as the barriers and facilitators to the improvement in the influenza vaccination coverage.

## 2. Methods

### 2.1. Study Areas

Zhejiang province is located at the east coastline of China, with a population of 60 million in 2024 and an area of 1055 KM^2^. The vaccination coverage of influenza vaccine was 3.2% in 2023. As in the rest of China, the vaccination clinics offered the influenza vaccine to workers of CHCs as the category II vaccine (self-paid vaccine), which needs payment for the cost of vaccine and the vaccination service by recipients. Most of the vaccination clinics were set in CHCs, and trained nursing workers were responsible for the vaccination. China’s Center for Disease Control and Prevention (CDC) encouraged influenza vaccination and defined the healthcare workers as one of the high-risk groups in the guidelines for influenza vaccination in China that is updated annually.

### 2.2. Study Design

We conducted a cross-sectional study from 1 to 31 April 2024 for all the workers of 22 CHCs in Zhejiang province. The anonymous questionnaire on a mobile phone APP was distributed by every surveyed CHC and was accessible to workers both at work and at home. Only one questionnaire would be completed by each respondent. The uniqueness of the questionnaire was identified by the cellphone number and the ID number of the workers. A reminder text message was sent once per week to the workers who had not finished the questionnaire. All data from the questionnaire survey were anonymized before the subsequent analysis process.

### 2.3. Sampling

Convenient sampling was applied in this study. First, one county/district each was chosen from 11 cities (there were 11 cities in total in Zhejiang province). Second, the CHC of the town where the county government was located was selected. Third, a CHC from the other town in the same county/district was selected. As such, there were 22 CHCs that were selected for this study in total.

### 2.4. Questionnaire

The questionnaire for this study was referred from a survey on medical residents in Italy [21] and modified based on the 3C model of vaccine hesitancy (complacency, confidence, convenience) [22]. The questionnaire also adjusted for application in a wider target group, as the socio-demographic backgrounds of the workers of CHCs varied, and all workers of CHCs were invited to participate in the investigation. Finally, there were nine sections in the questionnaire.

① Socio-demographics: sex; civil status; years of employment; profession classified as ”physicians”, “other healthcare workers” (i.e., nurses, technical laboratory assistants, technical radiology assistants), “administrative staff”, and “non-medical specialists” (i.e., pharmacologists); body mass index (BMI); and having children (yes/no).

② Influenza vaccination status: for the influenza season of 2020/2021, 2021/2022, and 2022/2023.

③ Rating for acceptance of influenza vaccination in the influenza season of 2022/2023, based on a 5-point Likert scale (strongly disagree, disagree, neither agree nor disagree, agree, and strongly agree).

④ Rating for refusal of influenza vaccination in the influenza season of 2022/2023, based on a 5-point Likert scale: strongly disagree, disagree, neither agree nor disagree, agree, and strongly agree.

⑤ Attitude on recommending influenza vaccination to patients, selecting from items such as: “Yes, in accordance with the guidelines of China CDC”, “Yes, based on the clinical experience”, “No, I would leave patients to decide for themselves”, “No, I discourage patients”, and “No, there was no chance to recommend in routine practice”.

⑥ Suggested strategies for improving influenza vaccination coverage among workers of CHCs, selecting multiple from items such as: “Vaccination for free of charge”, “Mandatory vaccination”, “Vaccination incentives”, “Training on influenza and vaccination in medical university”, “Improving availability for vaccination like more flexible opening times for vaccination clinics”, and “Other modes of immunization (e.g., nasal spray)”.

⑦ Source of information impacting the decision to accept/refuse vaccination: “None”, “Decree issued by the Ministry of Health”, “Mass media like newspapers, TV, internet, Mobile APP”, “Scientific publications”, “Information on institutional websites (China CDC etc.)”, and “Information received from workmates”.

⑧ Quality of information received: “Nil”, “Inadequate”, “Adequate”, “Good”, and “Excellent”.

⑨ Attitude toward preventive healthcare and a healthy lifestyle, including regular exercise, stop smoking and limit alcohol consumption, and vaccination for own child.

### 2.5. Statistical Analysis

Descriptive analyses were applied by calculating the mean with standard deviation (SD) for quantitative variables, median with interquartile range for ordinal variables, and absolute with frequency for qualitative variables. A bivariate analysis was conducted to detect the risk factors of the influenza vaccination status in the 2022/2023 season by using the chi-square test, Fisher’s exact test, or Kruskal–Wallis test, depending on the distributions of variables. The Bonferroni method was applied when the multiple comparison was conducted. Logistic regression analysis was applied to evaluate the association between the dependent variable (the influenza vaccination status in the 2022/2023 season) and all dependent predictors with a *p*-value < 0.10 in the bivariate analysis. The adjusted odds ratio (AOR) with the 95% confidence interval (CI) for each dependent predictor was also obtained. The multicollinearity was assessed using the variance inflation factor (VIF) before variables into the regression model. When a variable exceeded a VIF value of 5, it would be adjusted or eliminated if theoretically redundant. The statistical analyses were performed using Stata 15 software, and the significance level was set at 0.05.

## 3. Results

In total, 2205 of the 2511 workers contacted participated in this study, with a response rate of 87.81%. Table 1 presented the characteristics of the investigated workers and their influenza vaccination status in the 2022/2023 season. The influenza vaccination coverage in the 2022/2023 season was only 1.36%. Similar low coverage rates were observed in previous influenza seasons, with 1.29% for the 2020/2021 season and 1.39% for the 2021/2022 season, respectively.

Table 2 shows the attitude on accepting or refusing influenza vaccination in the 2022/2023 season by professional categories of workers of CHCs. The reasons for accepting the influenza vaccination similar in all professional categories included “To avoid spreading influenza among family”, “To avoid influenza”, “Influenza vaccination was strongly recommended from own CHC”, and “To avoid work absences”. For the other reasons, the level of agreement differed significantly between professional categories. The reason “To avoid influenza” met with the highest level of agreement while the reason “Influenza vaccination was strongly recommended by my own CHC” met with the lowest level of agreement in all professional categories. On the other hand, the level of agreement on reasons for refusing the influenza vaccination differed significantly among professional categories but were lowest for physicians for each item. Reasons like “Because of adverse reaction of previous influenza vaccinations”, “I did not have time”, and “I’m against vaccination generally” were more likely to be strongly disagreed upon among physicians. The reason “Not a high-risk group of influenza and possible complications” achieved the highest level of agreement, while the reason “I did not have time” achieved the lowest level of agreement in all professional categories.

**Figure 1:**
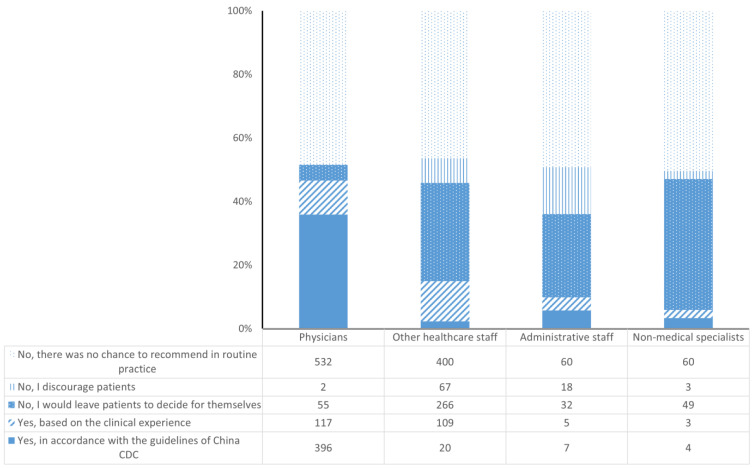
Recommendations of influenza vaccination by workers in community healthcare centers.

Of the respondents in the investigation, there were 1153 (52.29%) workers reporting having been consulted by patients for influenza vaccination advice in the 2022/2023 season, and the others stated “No, there was no chance to recommend in routine practice”. Of the subgroup who had been asked for advice on influenza vaccination, 37.03% stated “Yes, in accordance with the guidelines of China CDC” and 20.29% stated “Yes, based on the clinical experience”, while 34.87% stated ‘‘No, I would leave patients to decide for themselves” and 7.81% stated “No, I discourage patients”. Nearly 90% of the physicians recommended vaccination to their patients, but only 27.92% of the other healthcare workers, 19.35% of the administrative staff, and 11.86% of the non-medical specialists gave recommendations. On the other hand, patients were discouraged from influenza vaccination by 0.35% of the physicians, 14.50% of the other healthcare workers, 29.03% of the administrative staff, and 5.08% of the non-medical specialists, respectively (Figure 1).

Table 3 presented the socio-demographic and lifestyle factors related to the influenza vaccination status in the bivariate analysis, and variables with a *p*-value <0.10 were included in the next logistic regression analysis. VIF values for these variables ranged from 1.52 to 3.18. Therefore, no adjustment or elimination was performed for these variables.

The logistic regression analysis indicated that being a physician (AOR = 2.68, 95% CI:1.33–5.47) and BMI > 30 (AOR = 1.77, 95%CI:1.19–3.02) were positive factors significantly associated with receiving influenza vaccination, while regular exercise lowered the odds of being vaccinated (AOR = 0.71, 95%CI:0.52–0.91). The logistic regression also indicated that sources of information from the Ministry of Health (AOR = 1.59, 95%CI:1.07–2.65), scientific publications (AOR = 1.56, 95%CI:1.03–2.29), and institutional websites (AOR = 1.55, 95%CI:1.05–2.33) raised the odds of being vaccinated while information from mass media lowered the odds of being vaccinated (AOR = 0.37, 95%CI:0.21–0.58). “Recommendation on influenza vaccination to patients in accordance with the guidelines of China CDC” was significantly associated with receiving influenza vaccination (AOR = 5.58, 95%CI: 4.16–8.17) while “discourage patients for influenza vaccination” would lower the influenza vaccination coverage (AOR = 0.42, 95%CI: 0.11–0.75) (Table 4).

**Figure 2:**
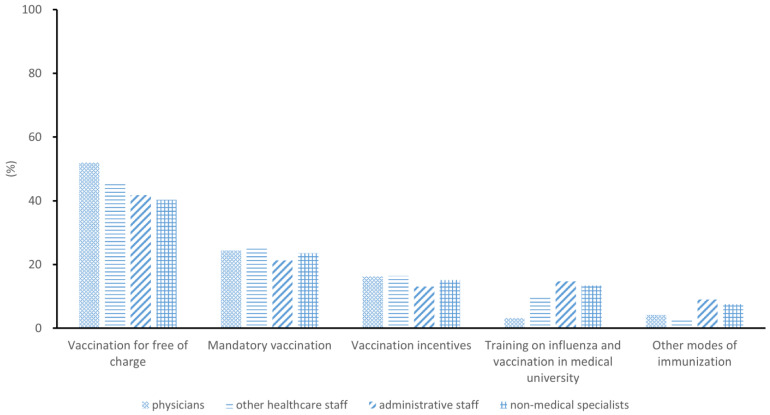
Suggested strategies for improving the influenza vaccination coverage from workers in community healthcare centers.

All professional groups stated that “vaccination for free of charge” would be the most appropriate strategy for improving the influenza vaccination coverage among workers of CHCs (40.34–52.00%), followed by “mandatory vaccination” (21.31–25.06%) (Figure 2).

## 4. Discussion

This study investigated the influenza vaccination status among workers of CHCs for the first time, as well as the attitude, barriers, and facilitators for influenza vaccination. The strengths of this study included the current influenza vaccination status among workers of CHCs and attitude on influenza vaccination and the study having a relatively large sample size with a high response rate. Our findings provided useful evidence on in influenza vaccination behaviors among workers of CHCs in Zhejiang province, China. This study could inform policy-makers to promote free influenza vaccination or mandatory influenza vaccination programs in workers of CHCs or other health promotion interventions targeting influenza prevention in the CHC work environment in China.

Workers in healthcare institutions are the priority group for influenza vaccination in China and abroad. However, this study observed a very low coverage of influenza vaccine in the investigated workers of CHCs in Zhejiang province. Socio-demographic, professional, lifestyle, and knowledge-associated factors emerged as determinants for their attitude toward influenza vaccination and vaccination status.

The findings of our investigation demonstrated that the coverage of influenza vaccine among workers of CHCs was less than 2% whether before or after the COVID-19 pandemic, which was lower than elsewhere in other areas of China and worldwide. A meta-analysis indicated that the overall influenza vaccination coverage among healthcare workers was 41.7%, and the specific coverage was 28.5% in Asia [23]. A report from Europe revealed that the influenza vaccination coverage was constantly around 10% from 2013–2016 [18], and another report indicated that the influenza vaccination coverage was over 20% in France and Spain [24]. Furthermore, Children’s Hospital of Henan province (China) found that the influenza vaccination rates among medical workers were 10.3% in 2018, before the COVID-19 pandemic [25]. During the COVID-19 pandemic (2019–2020), a web-based survey revealed that the influenza vaccination coverage among healthcare workers had been raised up to 67% under policy of free vaccination and workplace vaccination requirements [26]. From 2020 to 2022, Ma et al. found that the influenza vaccination coverage rates were 43.7% and 35.4% during the COVID-19 pandemic in the 2020/2021 and 2021/2022 seasons, respectively [27]. Obviously, the influenza vaccination coverage in our study was significantly lower, and we assumed there were several reasons. First, it might be related to the free influenza vaccination policy. Zhejiang province had neither offered free influenza vaccination nor introduced a mandatory influenza vaccination policy for medical workers. Second, the target interviewees of this study were workers of CHCs, including physicians and other non-medical workers that had a lower coverage. Third, the cultural background, health education, and personal awareness in the other investigation sites would be different from Zhejiang province, which should be clarified through comparative studies in future.

Physicians and other healthcare workers were observed as having higher influenza vaccination coverage than administrative or non-medical workers in our study—a finding consistent with the international literature [28,29]. Vaccine hesitancy among healthcare workers is a public health challenge [30]. However, this challenge would be more significant among non-medical workers of CHCs from our findings. The difference might be due to physicians and other healthcare workers having longer experience in the clinical field and easier access to scientific literature (especially for physicians), resulting in a heightened awareness and attention on influenza-related information and influenza vaccines. Another possible reason is that workers of CHCs working directly with patients had stronger awareness of reducing the risk of infection and nosocomial transmission of influenza.

For the refusal of influenza vaccination, we found some interviewees did not consider influenza as a threat to their health and judged influenza vaccination as unnecessary consequently. These findings were similar to previous reports, showing misconceptions of the risk of influenza infection among healthcare workers [13,30]. Our findings revealed a lack of correct awareness and attitude in workers of CHCs that medical workers were more exposed to influenza viruses because of their occupation. Furthermore, influenza vaccination for medical workers should be considered as an altruistic action to protect patients who could not be vaccinated for contradictions or other reasons or who were less effective for vaccination due to immunosuppression. Another worrying reason for refusing influenza vaccination was the personal conviction of anti-vaccines or anti-medications among all of the professional groups, which indicated that there was a proportion of workers of CHCs that had a mistrust of vaccines. A report from Europe demonstrated that lack of communication on side effects and perceived financial interests were the main reasons for vaccine hesitancy [31]. We suggested that influenza vaccination should be placed as one of the healthy lifestyles. Establishing the conviction of the importance of influenza vaccination for self-protection, protecting family members and patients should also be emphasized for driving vaccination among medical workers. The last reason for refusal of influenza vaccination among interviewees in this study was the safety concern on the previous influenza vaccinations. This response was similar to one study on influenza vaccine hesitancy among nurses, which indicated that those who refused vaccination wished to maintain a healthy status but perceived the vaccine as not protecting their health due to the poor effectiveness or that it could even harm their health from a potential adverse reaction or cause a disorder of the immune system [21]. Actually, the safety of the influenza vaccine had been proven, and most of the adverse reactions following influenza vaccine were mild, self-limiting, and resolving within 2 days. Disclosing the vaccine safety data would help to improve the confidence in the vaccine and to eliminate the vaccine hesitancy among medical workers.

An unexpected finding of this investigation was that a better lifestyle was generally related to the optimistic influenza vaccination status, expect for the “stop smoking and limit alcohol consumption” category, which showed no significant association with influenza vaccination. It was contrasted with previous reports showing a higher vaccination coverage and positive attitude on vaccination among persons with physical activity [29,32], as well as with a review that indicated that a lower BMI was a barrier to vaccination [14]. However, another study among older people from Brazil reported that a sedentary lifestyle was a constraint for influenza vaccination [33]. In the Chinese context, the possible explanation for our results was the complacency of the 3C model of vaccine hesitancy [22]. We assumed that worker of CHCs who had a healthy lifestyle or a lower BMI probably had self-confidence in their health status and had an opinion of low risk of infection against influenza, deeming that vaccination was not a necessary preventive action. A large-scale and rigorous investigation should be carried out to confirm the association between healthy lifestyle and influenza vaccination behavior among workers of CHCs.

The investigated workers of CHCs reported their information source of influenza or influenza vaccination. Except for mass media, other sources like the Ministry of Health, scientific publications, etc., had an effect on increasing the influenza vaccination coverage. Abramson et al. [29] had reported similar findings that mass media had a negative effect on healthcare workers’ decisions of receiving influenza vaccination. It was assumed that the accuracy and authority of information might not be as good as those from the government, specialist agencies, or academic literature. Notably, there was still a significant proportion of respondents who were short of information on influenza vaccination. We worried that these workers of CHCs might be uninfluenced by any type of information in making decisions on recommending vaccination to their patients or deciding whether or not to vaccinate themselves. Mass media has a wide range of audiences and many communicative channels, with it having a good advantage in communication efficiency [34]. Providing scientific, correct information or data in mass media accompanied by verifiable sources is necessary to improve, not only for the general population but also for the workers in medical institutions, their awareness of influenza to avoid misconceptions and deal with rumors, thus sustaining the personal motivation of receiving influenza vaccination [35].

For the recommendations on improving influenza vaccination coverage among workers of CHCs from this investigation, the free influenza vaccination policy and mandatory vaccination policy were most frequently suggested and had a high consistency among all professional categories. It was consistent with the recent reports from China, which indicated that the free policy and vaccination requirement on influenza vaccination could improve the coverage among healthcare workers in hospitals. During the 2019/2020 influenza season, a significant increase in influenza vaccination coverage up to 67% in healthcare workers was observed in a short term under a combination of policy of free vaccination and vaccination requirements of the influenza vaccine [26]. Children’s Hospital Affiliated to Zhengzhou University implemented a policy of free influenza vaccination for key department workers since 2020. A sharp increase in influenza vaccination coverage was observed from 11.6% in 2019 to 81.3% in 2022 [25]. In this investigation, the third best measure to raise the influenza vaccination coverage from interviewees’ mind was the incentive policy. As reported by a previous study, these incentives included monetary bonusing or giving a half-day holiday to individuals who received influenza vaccination [19].

This study has some limitations. First, although the response rate achieved 87.81%, the characteristics of non-responders could not be collected, which could not confirm whether there was difference in the distributions of socio-demographics between responders and non-responders. Second, all the data were self-reported in this study. Thus, the desirability and recall bias could not be avoided, though these biases were limited due to the anonymization process. Third, convenience sampling and the selection of only 22 CHCs may limit generalizability, and this gap could be addressed by a more rigorous study design in the future.

## 5. Conclusions

A poor compliance with influenza vaccination among workers of CHCs was observed, and it might be attributed to several risk factors; we found that being a physician, having no regular exercise habit, and receiving information on influenza vaccination from authoritative sources would raise the vaccination coverage among workers of CHCs. It was urgent to take actions on improving their understanding, awareness, and confidence on influenza vaccination, such as self-protection, reducing nosocomial infection and safety of vaccine. Free influenza vaccination and vaccination requirements policies might be helpful for enhancing vaccine uptake, especially for physicians and other healthcare workers, as they have a higher risk of exposure and a high probability of disease transmission.

## Figures and Tables

**Table 1:** Characteristics of the investigated workers of CHCs and influenza vaccination coverage of the 2022/2023 season.

Variable	Level	N	%	Vaccination Coverage (%, 95%CI)
Sex	Male	1101	49.93	1.36 (1.18–1.57)
	Female	1104	50.07	1.36 (1.19–1.54)
Civil status	Single	538	24.40	1.11 (1.05–1.33)
	Married	1397	63.36	1.43 (1.26–1.63)
	Divorced	239	10.84	1.25 (1.15–1.59)
	Widowed	31	1.41	3.22 (1.80–5.48)
Years of employment	0–9 yrs	486	22.04	1.23 (1.09–1.29)
	10–19 yrs	1423	64.54	1.40 (1.20–1.59)
	≥ 20 yrs	296	13.42	1.35 (1.12–1.54)
Profession	Physicians	1102	49.98	1.45 (1.25–1.88)
	Other healthcare workers	862	39.09	1.39 (1.18–1.79)
	Administrative staff	122	5.53	0.82 (0.45–1.05)
	Non-medical specialists	119	5.40	0.84 (0.36–1.17)
BMI	<18	752	34.10	1.19 (1.03–1.38)
	18–25	1106	50.16	1.35 (1.11–1.50)
	26–30	239	10.84	1.67 (1.37–2.16)
	>30	108	4.90	1.85 (1.32–2.63)
Have children	Yes	1502	68.12	1.46 (1.17–1.79)
	No	703	31.88	1.13 (1.05–1.26)

95%CI: 95% confidence interval.

**Table 2:** Distribution of attitudes on influenza vaccination in the 2022/2023 season by professional categories.

Attitude	Physicians *	Other Healthcare Workers *	Administrative Staff *	Non-Medical Specialists *	Total *	*p* #
Acceptance						
Consider themselves as a high-risk group	3.32 (4, 3)	2.82 (3, 4)	2.70 (3, 4)	3.05 (3, 4)	3.07 (3, 4)	0.04
To avoid spreading influenza among family	4.38 (5, 1)	4.17 (5, 1)	4.02 (5, 2)	3.88 (5, 2)	4.23 (5, 1)	0.13
To avoid spreading influenza among patients	4.36 (5, 1)	3.98 (5, 2)	3.05 (3, 3)	3.76 (5, 2)	3.95 (5, 2)	0.01
To avoid influenza	4.40 (5, 1)	4.38 (5, 1)	4.27 (5, 1)	4.28 (5, 1)	4.36 (5, 1)	0.85
Influenza vaccination was strongly recommended from own CHC	2.86 (3, 3)	2.75 (2, 2)	2.75 (3, 3)	2.07 (2, 2)	2.65 (3, 3)	0.33
To avoid work absences	3.91 (4, 2)	3.47 (4, 3)	3.72 (4, 2)	3.09 (3, 2)	3.88 (4, 2)	0.08
Scientific validity of the vaccine	4.18 (5, 1)	3.58 (4, 2)	3.33 (3, 2)	3.86 (5, 2)	3.82 (4, 2)	0.01
Refusal						
Does not consider influenza vaccine effective	2.20 (4, 1)	2.83 (4, 3)	2.67 (3, 4)	2.41 (3, 2)	2.56 (3, 3)	0.01
Does not consider influenza vaccine safe	1.97 (1, 3)	2.57 (2, 3)	2.67 (4, 3)	2.32 (2, 3)	2.68 (3, 3)	0.01
Influenza does not overweight the risk of vaccination	2.28 (2, 2)	2.92 (3, 2)	2.85 (3, 1)	2.57 (3, 3)	2.88 (4, 3)	0.01
Not a high-risk group of influenza and possible complications	3.38 (4, 2)	3.69 (4, 2)	4.11 (5, 2)	4.04 (4, 2)	3.72 (4, 1)	0.01
Not a high-risk group for spreading influenza in the general population	2.61 (3, 3)	3.07 (3, 1)	3.53 (4, 2)	3.38 (4, 2)	3.16 (3, 2)	0.01
Not a high-risk group for spreading influenza in patients	2.57 (3, 2)	3.08 (3, 2)	3.65 (4, 2)	3.63 (5, 2)	3.16 (3, 3)	0.01
Influenza vaccination was not recommended by own CHC	1.76 (1, 2)	1.97 (1, 2)	2.28 (2, 2)	2.03 (2, 2)	1.95 (1, 2)	0.01
Personal conviction to take less medications	2.70 (3, 3)	3.72 (4, 2)	3.77 (4, 2)	3.47 (4, 2)	3.55 (4, 3)	0.01
I’m against vaccination generally	1.35 (1, 0)	2.08 (1, 2)	2.37 (2, 2)	1.88 (1, 2)	2.13 (1, 2)	0.01
I forgot it	1.72 (2, 0)	2.31 (3, 2)	2.51 (3, 1)	2.05 (2, 0)	2.21 (3, 2)	0.01
I did not have time	1.29 (2, 1)	2.02 (1, 2)	1.8 (2, 0)	1.47 (2, 0)	1.52 (2, 1)	0.01
Because of adverse reaction of previous influenza vaccinations	1.13 (1, 0)	1.88 (3, 1)	2.17 (3, 2)	1.59 (2, 1)	1.90 (3, 1)	0.01

Note: *: median with interquartile range for 1–5-point Likert scale (1= strongly disagree, 2 = disagree, 3 = neither agree nor disagree, 4 =agree, 5 = strongly agree); #: *p*-value for the Kruskal–Wallis test.

**Table 3:** Bivariate analysis on socio-demographics and healthy lifestyle with influenza vaccination status in the 2022/2023 season.

Variable	Level	N	Received Vaccination	*p* for χ^2^-Test
Socio-demographics				
Sex	Male	1101	1.36	0.38
	Female	1104	1.36	
Civil status	Single	538	1.11	0.03
	Married	1397	1.43	
	Divorced	239	1.25	
	Widowed	31	3.22	
Years of employment	0–9 yrs	486	1.23	0.27
	10–19 yrs	1423	1.4	
	≥20 yrs	296	1.35	
Profession	Physicians	1102	1.45	0.01
	Other healthcare workers	862	1.39	
	Administrative staff	122	0.82	
	Non-medical specialists	119	0.84	
BMI	<18	752	1.19	0.02
	18–25	1106	1.35	
	26–30	239	1.67	
	>30	108	1.85	
Having children	Yes	1502	1.46	0.01
	No	703	1.13	
Regular exercise	Yes	1222	1.59	0.01
	No	983	1.15	
Stop smoking and limit alcohol consumption	Yes	305	2.26	0.01
	No	1900	1.29	
Vaccination for own child	Yes	2102	1.38	0.01
	No	103	1.02	
Sources of information				
Decree issued by the Ministry of Health	Yes	133	1.73	0.01
	No	2072	1.28	
Mass media	Yes	384	1.78	0.01
	No	1821	1.21	
Scientific publications	Yes	201	1.79	0.01
	No	2004	1.25	
Institutional websites	Yes	152	1.86	0.01
	No	2053	1.19	
Workmates	Yes	299	1.37	0.48
	No	1906	1.36	
Recommendation on influenza vaccination	Yes, in accordance with the guidelines of China CDC	427	1.79	0.01
	Yes, based on the clinical experience	234	1.41	
	No, I would leave patients to decide for themselves	402	1.37	
	No, I discourage patients	90	1.31	
	No, there was no chance to recommend in routine practice	1052	1.26	
Quality of information received	Nil	113	1.02	0.02
	Inadequate	187	1.05	
	Adequate	858	1.32	
	Good	917	1.37	
	Excellent	130	1.53	

**Table 4:** Logistic regression analysis for the dependent variable to influenza vaccination in the 2022/2023 season.

Variable	Level	AOR	95% CI	*p*
Socio-demographics				
Civil status	Single	Ref	-	-
	Married	1.12	0.53–1.35	0.24
	Divorced	0.82	0.45–1.16	0.31
	Widowed	1.32	0.43–3.93	0.18
Profession	Physicians	2.68	1.33–5.47	0.01
	Other healthcare workers	1.08	0.78–2.61	0.22
	Administrative staff	Ref	-	-
	Non-medical specialists	1.01	0.29–1.65	0.41
BMI	<18	1.02	0.61–4.22	0.37
	18–25	Ref	-	-
	26–30	1.19	0.88–1.81	0.29
	>30	1.77	1.19–3.02	0.01
Having children	Yes	1.29	0.91–1.88	0.11
	No	Ref	-	-
Regular exercise	Yes	0.71	0.52–0.91	0.01
	No	Ref	-	-
Stop smoking and limit alcohol consumption	Yes	1.06	0.89–1.25	0.26
	No	Ref	-	-
Vaccination for own child	Yes	1.03	0.74–2.06	0.47
	No	Ref	-	-
Sources of information				
Ministry of Health	Yes	1.59	1.07–2.65	0.02
	No	Ref	-	-
Mass media	Yes	0.37	0.21–0.58	0.01
	No	Ref	-	-
Scientific publications	Yes	1.56	1.03–2.29	0.02
	No	Ref	-	-
Institutional websites	Yes	1.55	1.05–2.33	0.01
	No	Ref	-	-
Recommendation on influenza vaccination	Yes, in accordance with the guidelines of China CDC	4.02	2.71–6.50	0.01
	Yes, based on the clinical experience	5.58	4.16–8.17	0.01
	No, I would leave patients to decide for themselves	1.37	0.95–2.27	0.38
	No, I discourage patients	0.42	0.11–0.75	0.01
	No, there was no chance to recommend in routine practice	Ref	-	-
Quality of information received	Nil	Ref	-	-
	Inadequate	0.88	0.31–1.82	0.52
	Adequate	1.02	0.42–2.22	0.38
	Good	1.04	0.47–1.89	0.51
	Excellent	1.22	0.32–2.17	0.62

AOR: adjusted odds ratio; 95%CI: 95% confidence interval.

## Data Availability

All data included in this study are available upon request by contact with the corresponding author.
